# Can Serum Drops Containing Doxycycline Provide a Supplemental Anti-Bacterial Effect in the Treatment of Bacterial Keratitis?

**DOI:** 10.3390/antibiotics12071145

**Published:** 2023-07-03

**Authors:** David Mora-Boellstorff, Kanwal Matharu, Vishal Jhanji, Regis P. Kowalski

**Affiliations:** The Charles T. Campbell Ophthalmic Microbiology Laboratory, Department of Ophthalmology, School of Medicine, University of Pittsburgh, Pittsburgh, PA 15213, USA

**Keywords:** doxycycline, bacterial keratitis, autologous serum drops, susceptibility

## Abstract

Purpose: Systemic doxycycline has been prescribed to reduce inflammation and enhance corneal healing in bacterial keratitis. Topical autologous serum drops (ASD) containing doxycycline following oral supplementation may additionally confer an anti-bacterial effect. The potential of this supplementation was evaluated by determining the in vitro susceptibility of bacterial keratitis isolates to doxycycline. Methods: The minimum inhibitory concentrations (MICs) of doxycycline against 100 bacterial keratitis isolates were determined using Etests. Twenty-seven *Staphylococcus aureus*, ten coagulase-negative *Staphylococci*, six *Streptococcus pneumoniae*, seven viridans group streptococci, seven other Gram-positive bacteria, nineteen *Pseudomonas aeruginosa*, eight *Serratia marcescens*, four *Moraxella* spp., two *Haemophilus* spp., and ten other Gram-negative bacteria isolates were tested. MICs of doxycycline were compared to a serum standard concentration of doxycycline (SSCD) of 4 µg/mL and concentrations that would be found in 50% and 20% serum component clinical preparations of ASD, corresponding to 50% SSCD (2 µg/mL) and 20% SSCD (0.8 µg/mL), respectively. MICs equal to or less than these values were used to deem a bacterial isolate susceptible. Results: For Gram-positive bacteria, susceptibilities to SSCD, 50% SSCD, and 20% SSCD were 86%, 65%, and 60%, respectively. For Gram-negative bacteria, susceptibilities to SSCD, 50% SSCD, and 20% SSCD were 37.2%, 23.3%, and 11.6%, respectively. Chi-squared analyses comparing Gram-positive and Gram-negative susceptibilities showed significantly greater susceptibility of Gram-positive bacteria at all three tested MICs (<0.0001, <0.0001, <0.0001). Conclusions: Our data suggest that autologous serum drops containing theoretic concentrations of doxycycline may provide an additional anti-bacterial effect in the treatment of bacterial keratitis, especially for Gram-positive bacterial keratitis compared to Gram-negative bacterial keratitis.

## 1. Introduction

The cornea specialist or comprehensive ophthalmologist has an arsenal of tools at their disposal in the treatment of bacterial keratitis, including topical antibiotics, oral antibiotics, vitamin C, autologous serum drops, amniotic membrane transplantation, and therapeutic keratoplasty. A combination of these tools is usually necessary for vision-threatening bacterial keratitis. Even so, bacterial keratitis is still a major contributor to visual morbidity. Infectious keratitis from any pathogen has an estimated incidence of 2.5–799 cases per 100,000 population/year worldwide, with an estimated 86–92% of those cases stemming from a bacterial cause in North America [1]. Developed countries tend to have fewer cases of infectious keratitis overall but a higher propensity for bacterial agents secondary to more prevalent contact lens use. Developing countries have more cases of infectious keratitis but a lower incidence of bacterial origin [1].

Oral doxycycline is frequently prescribed as an adjunct to topical antibiotics in the treatment of bacterial keratitis for its anti-inflammatory properties, with little recognition of any anti-bacterial contribution. Doxycycline inhibits key mediators of tissue remodeling throughout the body and consequently reduces the likelihood of visually devastating keratolysis [2,3,4,5,6].

Autologous serum drops (ASD) can be prescribed to promote corneal healing in bacterial keratitis. Growth factors, vitamin A, fibronectin, and lysozyme found in natural tears can be found in higher concentrations in patient-derived serum [7,8,9]. ASD can also be used to stabilize the ocular surface and promote corneal healing in non-infectious etiologies, such as neurotrophic epithelial defects, severe dry eye syndrome, Stevens–Johnson syndrome, limbal stem cell deficiency, ocular cicatricial pemphigoid, or other conjunctival cicatrizing diseases. If ASD are prepared following oral supplementation of doxycycline, it logically follows that doxycycline found in the patient’s serum will also be present in the patient’s ASD.

We speculate that some patients who receive oral doxycycline prior to receiving ASD may experience a dual benefit from the anti-inflammatory and anti-bacterial properties of doxycycline. After oral administration and absorption in the GI tract, a serum concentration of doxycycline will subsequently be present in the patient’s ASD. Frequently, corneal specialists will prescribe 50 or 100 mg of oral doxycycline twice daily in patients with corneal thinning. Pharmacokinetic studies of doxycycline have shown that for a multiple-dosage regimen of 200 mg/day, the mean serum concentration varies from 4.0 to 4.4 μg/mL [10]. Peak concentration occurs 2–4 h following ingestion [10,11]. However, several factors influence the absorption and subsequent serum concentration at any given time, including timing and macronutrient content of consumed meals, length of treatment, presence of loading dose, and rate of renal and gastrointestinal excretion. Given those variables, prior to identifying actual concentrations of doxycycline in ASD, we want to establish if doxycycline can provide an anti-bacterial effect in bacterial keratitis by evaluating minimum inhibitory concentrations (MICs) of doxycycline against bacterial isolates from cases of clinically significant keratitis. Although the MICs of doxycycline against many bacterial isolates have been described in the literature, this is the first study that seeks to identify MICs of doxycycline against specific bacterial strains known to have caused clinically significant keratitis.

## 2. Materials and Methods

**Figure 1:**
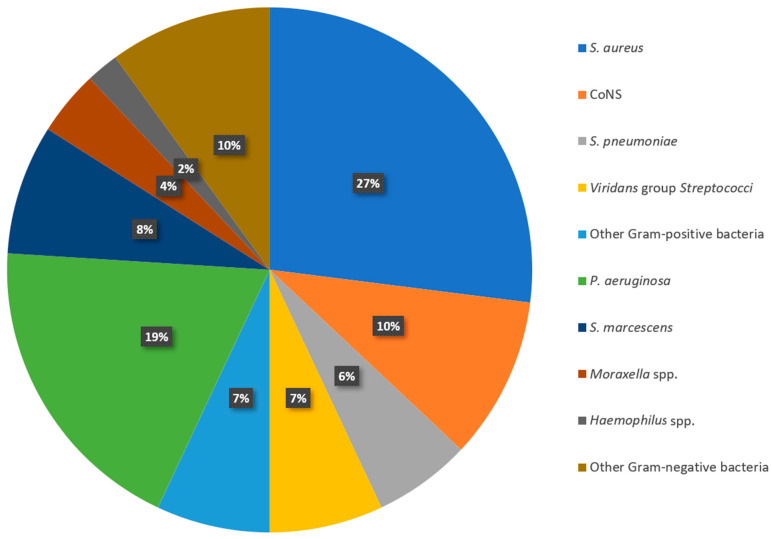
Percent incidence of bacteria isolated from keratitis from 1993 to 2018 (n = 2139) used as a reference for 100 bacterial keratitis isolates selected in this study.

One hundred de-identified bacteria isolated from keratitis were retrieved from the clinical tissue bank of the Charles T. Campbell Microbiology Laboratory at the University of Pittsburgh Medical Center. The isolates are stocked for validation testing of new anti-infectives and data monitoring for laboratory accreditation. Historical data of bacterial keratitis isolates from the Charles T. Campbell Microbiologic Laboratory were used to create a species-representative sample of 100 bacterial keratitis isolates [12]. We tested 27 (27%) *Staphylococcus aureus* (*S. aureus*), 10 (10%) coagulase-negative *Staphylococci* (CoNS), 6 (6%) *Streptococcus pneumoniae* (*S. pneumoniae*), 7 (7%) viridans group *Streptococci*, 7 (7%) other Gram-positive bacteria (3 *Enterococcus* spp., 2 *Diphtheroid* spp., 1 Group A *Streptococcus* spp., and 1 beta-hemolytic *Streptococcus* species), 19 (19%) *Pseudomonas aeruginosa* (*P. aerugionasa*), 8 (8%) *Serratia marcescens* (*S. marcescens*), 4 (4%) *Moraxella* spp., 2 (2%) *Haemophilus* spp., and 10 (10%) other Gram-negative bacteria isolates (3 *Proteus* spp., 2 *Escherichia coli*, and 1 each of *Acinetobacter* spp., *Klebsiella pneumoniae*, *Stenotrophomonas maltophilia*, *Brevundimonas* spp., and *Enterobacter cloacae*). Figure 1 depicts the percent incidence of bacteria isolated from keratitis from 1993 to 2018 (n = 2139).

MICs using Etests (Liofilchem, Abruzzi, Italy) were used to assess susceptibility to doxycycline. The concentration range of testing was from 256 to 0.016 µg/mL. For testing, bacterial isolates were retrieved from a −80 °C freezer and thawed at room temperature. The bacteria were streaked on either trypticase soy agar with 5% sheep blood or chocolate agar for *Haemophilus* spp. The streaked bacteria were incubated overnight in a 6% carbon dioxide (CO_2_) incubator at 37 °C. After incubation, bacteria were streaked a second time on the appropriate agar for another 24 h to ensure viability, identification, and purity. Each bacterial specimen was then suspended in 5 mL of trypticase broth to a 0.5 McFarland standard. Mueller–Hinton agar, Mueller–Hinton with blood agar, and chocolate plates (for *Haemophilus* spp.) were used as solid agar for susceptibility testing. A soft-tip cotton applicator was moistened with the bacterial suspensions and streaked across a quarter of an agar plate for a total of four separate bacterial isolates per agar plate. The two *Haemophilus* spp. isolates were streaked on half of a chocolate agar plate, respectively. Once streaked, the plates were allowed to dry for 10 min. A doxycycline Etest strip containing a decreasing antibiotic gradient from 256 μg/mL to 0.016 μg/mL was laid over the bacterial streak. The plates were placed in a 6% CO_2_ incubator at 37 °C for 24 h. After 24 h, the zones of inhibition were interpreted. MICs of doxycycline were compared to a serum standard concentration of doxycycline (SSCD) of 4 µg/mL and concentrations that would be found in 50% and 20% serum component clinical preparations of ASD, corresponding to 50% SSCD (2 µg/mL) and 20% SSCD (0.8 µg/mL), respectively. MICs equal to or less than these values were used to deem a bacterial isolate susceptible.

## 3. Results

**Figure 2:**
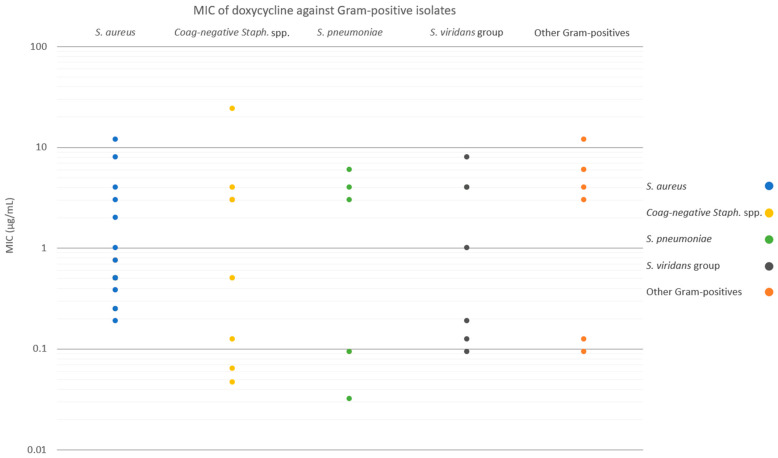
MICs of doxycycline against Gram-positive isolates, categorized by species.

**Figure 3:**
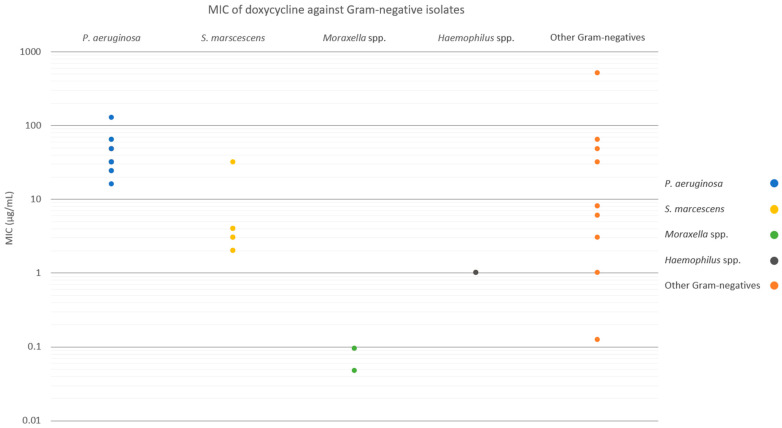
MICs of doxycycline against Gram-negative isolates, categorized by species. The MIC of a single Gram-negative isolate (*E. coli*) was greater than 256 µg/mL and was designated as 512 µg/mL.

**Figure 4:**
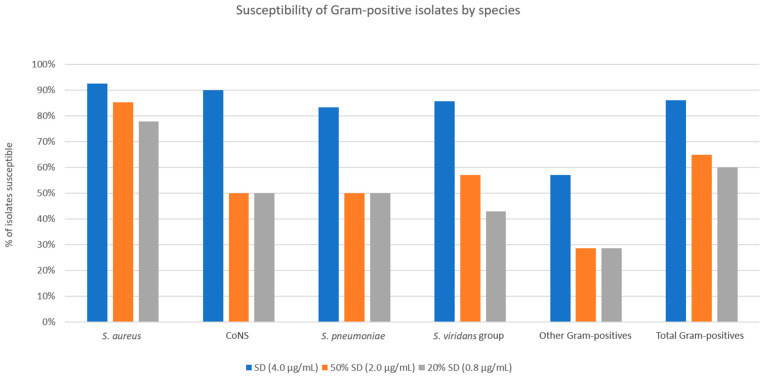
Percentage of Gram-positive isolates susceptible at three tested threshold concentrations of doxycycline, categorized by species.

Figure 2 and Figure 3 show the MICs of doxycycline against Gram-positive and Gram-negative isolates, respectively. For the entire Gram-positive isolate cohort, susceptibilities to SSCD, 50% SSCD, and 20% SSCD were 86.0%, 65.0%, and 60.0%, respectively (Figure 4). For *S. aureus* isolates, susceptibilities to SSCD, 50% SSCD, and 20% SSCD were 92.6%, 85.2%, and 77.8%, respectively. For coagulase-negative *Staphylococcus* spp., susceptibilities to SSCD, 50% SSCD, and 20% SSCD were 90.0%, 50.0%, and 50.0%, respectively. For *S. pneumoniae* isolates, susceptibilities to SSCD, 50% SSCD, and 20% SSCD were 83.3%, 50.0%, and 50.0%, respectively. For viridans group *Streptococci*, susceptibilities to SSCD, 50% SSCD, and 20% SSCD were 85.7%, 57.1%, and 42.9%, respectively. For other Gram-positive bacterial isolates (three *Enterococcus* spp., two *Diphtheroid* spp., one Group A *Streptococcus* spp., and one beta-hemolytic *Streptococcus* spp.), susceptibilities to SSCD, 50% SSCD, and 20% SSCD were 57.1%, 28.6%, and 28.6%, respectively. Notably, for the Gram-positive isolate cohort, excluding *S. aureus*, susceptibilities to SSCD, 50% SSCD, and 20% SSCD were 80.0%, 46.7%, and 43.3%, respectively.

**Figure 5:**
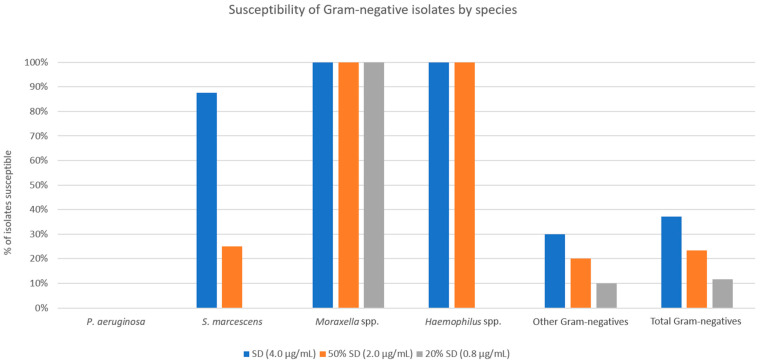
Percentage of Gram-negative isolates susceptible at three tested threshold concentrations of doxycycline, categorized by species.

For the overall Gram-negative isolate cohort, susceptibilities to SSCD, 50% SSCD, and 20% SSCD were 37.2%, 23.3%, and 11.6%, respectively (Figure 5). All *P. aeruginosa* isolates were resistant to all three threshold concentrations tested. For *S. marcescens*, susceptibilities to SSCD, 50% SSCD, and 20% SSCD were 87.5%, 25.0%, and 0.0%, respectively. All *Moraxella* spp. isolates were susceptible at all threshold concentrations tested. For *Haemophilus* spp., susceptibilities to SSCD, 50% SSCD, and 20% SSCD were 100%, 100%, and 0.0%, respectively. For other Gram-negative isolates (three *Proteus* spp., two *Escherichia coli*, and one each of *Acinetobacter* spp., *Klebsiella pneumoniae*, *Stenotrophomonas maltophilia*, *Brevundimonas* spp., and *Enterobacter cloacae*) susceptibilities to SSCD, 50% SSCD, and 20% SSCD were 30.0%, 20.0%, and 10.0%, respectively.

**Figure 6:**
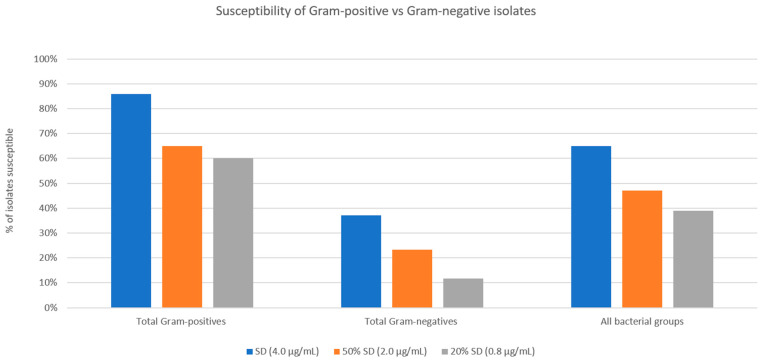
Percentage of Gram-positive isolates, Gram-negative isolates, and all bacterial isolates susceptible at three tested threshold concentrations of doxycycline. Gram-positive isolates showed a higher percentage of susceptibility at all three tested concentrations compared to Gram-negative isolates.

A comparison of the performance of the Gram-positive isolate cohort to the Gram-negative isolate cohort is shown in Figure 6. Chi-squared analyses comparing Gram-positive and Gram-negative susceptibilities at all three threshold MICs showed significantly higher percentages of susceptible Gram-positive bacteria (<0.00001 for SSCD, <0.000036 for 50% SSCD, and <0.00001 for 20% SSCD).

## 4. Discussion

Doxycycline, a tetracycline antibiotic, inhibits the function and synthesis of the matrix metalloproteinase (MMP) family [3,4] by chelating ions that are vital for their functioning. Matrix metalloproteinases are enzymes that catalyze the proteolysis of ECM proteins with the assistance of a metal ion. By cleaving protein components of the ECM, MMPs are heavily involved in a host of activities surrounding the process of tissue remodeling, including cellular differentiation, proliferation, migration, angiogenesis, and apoptosis—all broad components of the body’s inflammatory response [2]. MMPs have been shown to promote corneal epithelial basement membrane destruction, facilitating stromal ulceration [13]. Doxycycline additionally inhibits inflammation and wound healing by inhibiting IL-1 synthesis, a cytokine that modulates both MMP expression by corneal stromal fibroblasts and MMP synthesis within keratocytes, regulates keratocyte apoptosis during corneal wound healing, and upregulates keratocyte growth factor in corneal fibroblasts [5,14,15,16]. The counteracting forces of extracellular matrix (ECM) deposition and degradation maintain the integrity of the corneal stroma, and whenever this balance favors degradation, stromal ulceration occurs [13,17]. In bacterial keratitis, a robust accompanying inflammatory response from MMPs and collagenases can result in rapid corneal melting and corneal perforation.

Additionally, doxycycline selectively inhibits bacterial protein synthesis by blocking aminoacyl transfer RNA from the acceptor site of the bacteria-specific 30S ribosomal subunit-mRNA complex. By preventing transfer RNA from binding messenger RNA, protein translation is disrupted, and cellular dysfunction and apoptosis ensue. Doxycycline’s anti-inflammatory and anti-bacterial properties give it the potential to have an additive beneficial effect in the treatment of bacterial keratitis via both mechanisms.

Animal studies, case reports, anecdotal evidence, and knowledge of the mediators of corneal stromal remodeling in inflammatory processes form the basis for the use of doxycycline to treat bacterial keratitis [17,18]. McElvanney described two patients with contact lens-associated *Pseudomonas* keratitis with corneal melting that stabilized with treatment with systemic doxycycline (100 mg twice daily), along with topical treatment with ofloxacin and ceftazidime [17]. Given the high MIC required to inhibit *Pseudomonas aeruginosa* both in our study (MIC 41.3) and in the literature (MIC > 32), it is appropriate to hypothesize that doxycycline’s main contribution in *Pseudomonas* keratitis is to reduce inflammation rather than provide a direct anti-bacterial effect [19,20]. Systemic doxycycline has also been shown in rabbit models to reduce the progression of corneal melting to corneal perforation in *Pseudomonas* keratitis by 50% [18].

Golub et al. established that the anti-inflammatory and anti-bacterial components of tetracycline act independently by removing the dimethylamino group at C4, inactivating its anti-bacterial properties and creating a “chemically modified non-antimicrobial” tetracycline analog [21]. This chemically modified non-antimicrobial analog of tetracycline was shown to preserve MMP inhibition in extracts of human rheumatoid synovial tissue and rat bones [22]. However, there are no randomized controlled trials in humans showing an improvement in visual outcomes of bacterial keratitis with systemic doxycycline. Additionally, there are no studies of any type describing the use of topical doxycycline in bacterial keratitis.

Overall, Gram-positive bacteria showed relatively higher levels of susceptibility to theoretic concentrations of doxycycline than would be found in typical ASD preparations. *S. aureus* is a common culprit of ocular and periocular infections, including blepharitis, dacryocystitis, conjunctivitis, keratitis, and endophthalmitis. Up to 35% of the general population is believed to be colonized with the bacterium [23]. Notably, of the Gram-positive isolates tested in this experiment, *S. aureus* had the highest percentage of susceptible isolates at 50% SSCD and 20% SSCD (85.2% and 77.8%, respectively). An additive effect between the anti-bacterial and anti-inflammatory properties of doxycycline could explain the particular success of doxycycline against *S. aureus*, as *S. aureus* has been shown to induce increased expression of multiple MMPs in human dermal and synovial fibroblasts [24]. When excluding *S. aureus*, the remaining Gram-positive isolates were 47% and 43% susceptible at 50% SSCD and 20% SSCD, respectively. Coagulase-negative *Staphylococcus* has been identified with great frequency on the ocular surface, but its role as a commensal organism versus a pathogen has yet to be firmly established [25,26]. Doxycycline produced more modest inhibition of coagulase-negative *Staphylococcus* at the clinically relevant 50% SSCD and 20% SSCD, with only 50% of isolates susceptible at both concentrations. A similar pattern of more modest inhibition was seen among the other Gram-positive bacteria tested in this study (Figure 4). The robust inhibition of *S. aureus* growth compared to other Gram-positive bacteria suggests a strong supplementary anti-bacterial effect of doxycycline against *S. aureus* infection.

Compared to Gram-positive isolates, Gram-negative isolates showed a significantly lower percentage of susceptible strains at concentrations of doxycycline that would be found in standard preparations (50% SSCD and 20% SSCD found in 50% and 20% ASD, respectively). However, a few species showed higher rates of susceptibility compared to other Gram-negative species. All *Moraxella* spp., *Haemophilus* spp., *Acinetobacter* spp., and *Brevundimonas* spp. isolates were susceptible at 50% SSCD and 20% SSCD. Doxycycline has a broad spectrum of coverage against Gram-positive and Gram-negative bacteria but historically does not have good anti-pseudomonal coverage. The average MICs of *P. aeruginosa* obtained in our study (41.3) were similar to those found in the literature (>32) [19,20]. The high prevalence of *P. aeruginosa* among Gram-negative bacterial keratitis (19/50 isolates in our sample) is likely a large contributor to the statistical difference seen between the percentage of susceptible Gram-positive vs. Gram-negative isolates.

*Moraxella* spp. are aerobic, Gram-negative rods that are typically less fulminant than other Gram-negative bacterium, but can nevertheless cause prolonged, persistent bacterial keratitis with devastating ocular manifestations. Classically associated with angular blepharoconjunctivitis, *Moraxella* has also been identified as a causative pathogen in keratitis with hypopyon, significant stromal ulceration, corneal thinning, and perforation eventually requiring therapeutic keratoplasty [27,28,29,30]. Tan et al. (2017) showed that *Moraxella* keratitis demonstrated an increasing incidence over a 12-year span at a UK tertiary hospital, accompanied by an increase in Gram-negative, fungal, and acanthamoeba keratitis with a corresponding decrease in Gram-positive keratitis over that same time period [31]. In a case series by Das et al., 46% of patients presented with hypopyon, which was associated with worse visual outcomes in a separate investigation of *Moraxella* keratitis by McSwiney et al. [32]. Slower epithelial healing time has also been noted in *Moraxella* keratitis [29,30,32]. As a result, eyes with *Moraxella* keratitis are at a high risk of corneal perforation and frequently require surgical management to pre-empt or respond to this complication. As many as 46–51% of *Moraxella* keratitis cases require surgical intervention [28,29,30,33] and often result in poor visual outcomes, with 26–51% of patients having visual acuity of counting fingers or less despite aggressive intervention [29,30,32]. In cases of severe *Moraxella* keratitis, oral doxycycline has been used for its conventional indication to reduce corneal thinning via its anti-inflammatory effects [34]. As of this writing, there are no studies evaluating the anti-bacterial impact of doxycycline, oral or topical, in *Moraxella* keratitis. Our experimental study shows that doxycycline found in ASD could provide a promising anti-bacterial adjunct to the current standard of care for *Moraxella* keratitis.

Our study suggests that theoretical concentrations of doxycycline that could be found in routinely used preparations of ASD may exceed the MIC necessary to provide an anti-bacterial effect in the treatment of bacterial keratitis that would be additive to the anti-inflammatory properties described earlier in this discussion. In particular, Gram-positive infections and, notably, *Moraxella* keratitis showed in vitro inhibition to 50% and even 20% concentrations of doxycycline.

Limitations of this study include an assumption of doxycycline found in preparations of ASD following oral supplementation [10,35]. As mentioned in the introduction, several factors, including the timing of blood sampling following oral dosing, presence of loading dose, meal timing, and content, as well as the rate of gastrointestinal excretion, can cause inter-patient variability of serum doxycycline concentration. Additionally, it is assumed that the concentration of doxycycline within the serum is perfectly maintained during the creation of ASD; it will be necessary to measure the concentrations of doxycycline in the serum and in subsequent serum tears to know how much doxycycline survives the production process. For these reasons, actual concentrations found in multiple ASD preparations may vary from those assumed in this study. We recognize that identifying actual concentrations of doxycycline in ASD in a future study is paramount to understanding the actual anti-bacterial effect that doxycycline in ASD might provide. Additionally, the cohort of 100 bacterial isolates was selected as a representative sample of keratitis isolates submitted to the Charles T. Campbell Microbiologic Laboratory associated with a tertiary academic institution and may not be generalizable to the true incidence of bacterial keratitis in the broader population. Additionally, there is some debate about the use of ASD in cases of infectious keratitis, as some believe that the same growth factors that encourage corneal re-epithelization may provide pathogens with macromolecules that facilitate survival.

To conclude, this study supports the concept that if doxycycline exists at certain concentrations in ASD following oral supplementation, doxycycline could potentially confer an anti-bacterial effect. Further studies are needed to evaluate the concentration of doxycycline within ASD. Additionally, we cannot ascertain that this effect would be clinically relevant. Subsequent studies comparing outcomes of bacterial keratitis with oral supplementation of doxycycline before and after the creation of ASD will be required to support that claim.

## Data Availability

The data presented in this study are available in this article.
